# Mathematical modeling of mass and energy transport for thermoembolization

**DOI:** 10.1080/02656736.2020.1749317

**Published:** 2020

**Authors:** David Fuentes, Samuel J. Fahrenholtz, Chunxiao Guo, Christopher J. MacLellan, Rick R. Layman, Beatrice Rivière, R. Jason Stafford, Erik Cressman

**Affiliations:** a Department of Imaging Physics, Anderson Cancer Center, The University of Texas MD, Houston, TX, USA; b Department of Computational and Applied Mathematics, Rice University, Houston, TX, USA; c Department of Interventional Radiology, Anderson Cancer Center, The University of Texas MD, Houston, TX, USA

**Keywords:** Bioheat transfer, finite elements, embolization, hepatocellular carcinoma, transcatheter chemistry

## Abstract

**Background:**

Thermoembolization presents a unique treatment alternative for patients diagnosed with hepatocellular carcinoma. The approach delivers a reagent that undergoes an exothermic chemical reaction and combines the benefits of embolic as well as thermal- and chemical-ablative therapy modalities. The target tissue and vascular bed are subjected to simultaneous hyperthermia, ischemia, and chemical denaturation in a single procedure. To guide optimal delivery, we developed a mathematical model for understanding the competing diffusive and convective effects observed in thermoembolization delivery protocols.

**Methods:**

A mixture theory formulation was used to mathematically model thermoembolization as chemically reacting transport of an electrophile, dichloroacetyl chloride (DCACl), within porous living tissue. Mass and energy transport of each relevant constituent are considered. Specifically, DCACl is injected into the vessels and exothermically reacts with water in the blood or tissue to form dichloroacetic acid and hydrochloric acid. Neutralization reactions are assumed instantaneous in this approach. We validated the mathematical model predictions of temperature using MR thermometry of the thermoembolization procedure performed in *ex vivo* kidney.

**Results:**

Mathematical modeling predictions of tissue death were highly dependent on the vascular geometry, injection pressure, and intrinsic amount of exothermic energy released from the chemical species, and were able to recapitulate the temperature distributions observed in MR thermometry.

**Conclusion:**

These efforts present a first step toward formalizing a mathematical model for thermoembolization and are promising for providing insight for delivery protocol optimization. While our approach captured the observed experimental temperature measurements, larger-scale experimental validation is needed to prioritize additional model complexity and fidelity.

## 1. Introduction

Hepatocellular carcinoma (HCC) has a high mortality rate and the yearly death rate nearly equals the incidence of roughly 1 million per year [1,2]. A partial hepatectomy to remove the disease is the optimal treatment and is potentially curative. However, the potential population for this ideal surgical approach is small, ≈5% in the US [3–5]; HCC is more frequently diagnosed in later stages of the disease because of a general lack of characteristic symptoms during early stages of the disease [6,7]. Ablation and embolization are the two most common minimally invasive methods used in treating unresectable HCC [8]. These are established therapies with a known survival advantage [9,10]. Thermally ablative therapy, including radiofrequency ablation, microwave ablation, high-intensity focused ultrasound ablation, cryotherapy, and laser ablation, utilizes thermal energy to destroy the disease. Embolization techniques utilize a direct injection of a chemotherapeutic agent or radiation into the hepatic artery, with or without ethiodized oil (Lipiodol^[*textregistered*]^) and a procoagulant material to promote intratumoral retention. Unfortunately, incomplete ablation is more prevalent than commonly believed [11, 12]. Total necrosis rates are less than 50% of treated nodules. Further, residual tumor is present in up to 90% of nodules treated with embolization [13]. HCC is typically a highly vascularized lesion with multiple blood supplies, and insufficient hypoxia conditions from partial embolization commonly lead to treatment failure. Recurrences are frequent [14,15], and incomplete treatment can incite more aggressive tumor behavior [16–18]. There is a well-recognized need for novel methods that can intricately balance treatment of the disease extent with preservation of liver function and minimal risk of recurrence and metastasis.

Thermoembolization [19,20] represents a novel treatment approach for patients battling HCC. Thermoembolization is a procedure in which an acid chloride reagent is delivered by an endovascular route. Water in the surrounding tissue hydrolyzes the acid chloride and causes a strong exothermic reaction. This approach is unique and combines the benefits of embolic as well as thermal- and chemical-ablative therapy modalities. Several studies have also considered a sequential embolization and ablation approach [21–23]. However, thermoembolization combines the multiple therapies into a single procedure. The solution of acid chloride dissolved in an oily solvent is delivered through a small catheter into the target artery. The oily solvent delays reactions and provides time for the acid chloride to reach the target tissue. Once released, the acid chloride reacts vigorously with any water or available functional groups present in the tissue and simultaneously generates an acidic local environment. This exothermic hydrolysis of acid chloride offers multiple mechanisms of local tissue destruction based on the distribution of the resulting heat and reaction byproduct. The target tissue and vascular bed are subjected to simultaneous hyperthermia, ischemia, and chemical denaturation. Intuitively, embolic effects of this technique reduce blood flow near the treatment zone and thus can reduce major heat-sink limitations observed with ablation of liver tissue. Selective delivery to the target tumor is achieved through vessel selection. Further, inflammation in the periphery of the treatment zone can enhance delivery of chemical denaturant byproducts that may synergistically increase the diameter of the lethal zone of this therapy. A relatively high concentration of these reaction byproducts is left behind in a localized region of the devascularized treatment area and serves as a local diffusion reservoir of chemical denaturant to decrease risk of local recurrence, which is a common problem with purely thermal ablation techniques.

Current efforts are promising [19,20] and have demonstrated a 40:1 ratio of coagulated tissue volume to injected material and up to a 24.1 °C temperature rise. However, from a clinical standpoint, this potential for extensive tissue damage suggests a need for greater understanding of the behavior of these materials when delivered by the endovascular route. Suboptimal delivery procedures may falsely lead to inferior results with this technique. We, therefore, sought to develop a mathematical modeling approach toward understanding the competing diffusive and convective effects observed in thermoembolization delivery procedures.

High-fidelity mathematical modeling of thermoembolization is challenging and involves chemically reacting multicomponent flows within porous living tissue. Current mathematical models in the literature approach exothermic chemical reactions in tissue as a single parabolic equation with a heat source forcing function [24]. Efficient and stable numerical methods based on conservation laws are fundamental toward understanding this novel therapeutic approach. In particular, mixture theory formulations provide an axiomatic framework for development of our approach. Mixture theory appears in the mathematics, aerospace, biomedical, geosciences, and petroleum engineering literature [25–28]. Each field has slightly different notation and considerations specific to the target application area. Generally, multiphase oil and gas flow [25] applications and multiphase liquid-vaporsalt flow geothermal applications [26,29] within the porous seafloor assume immiscible or miscible Darcy flow. Here we coupled Darcy flow models to energy conservation principles to model mass transport and heat transfer in biological tissues [30–36].

## 2. Methods

### 2.1. Numerical methods

**Figure 1.**
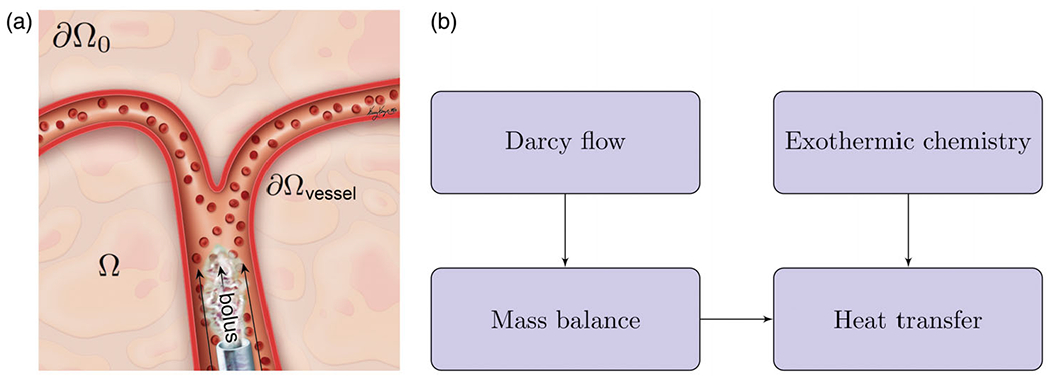
Overview of numerical approach. (a) A bolus of acid chloride and oily solvent is delivered through the blood vessels. The bolus enters the liver parenchyma, Ω, through the tissue-vessel interface, $\partial \Omega_{\text{vessel}}$. (b) The injection pressure drives Darcy flow through the porous liver tissue. Mass balance is used to track the bolus transport. Heat transfer is a combination of both convection of the chemically reacting acid chloride and heat diffusion. Atmospheric pressure and zero flux are assumed on the far boundary, $\partial \Omega_{0}$.

An overview of our numerical approach is presented in Figure 1. For a general chemically reacting flow through vessels into porous tissue, we must consider displacement of blood by the injected bolus of acid chloride and oily solvent in a porous tissue $\Omega \subset R^{3}$ over a time period [0, *T*]. Specifically, we consider the hydrolysis of dichloroacetyl chloride (DCACl). This reaction generates dichloroacetic acid (DCA) and hydrochloric acid (HCl) as well as releases substantial heat energy (93 kJ/mole), as follows:


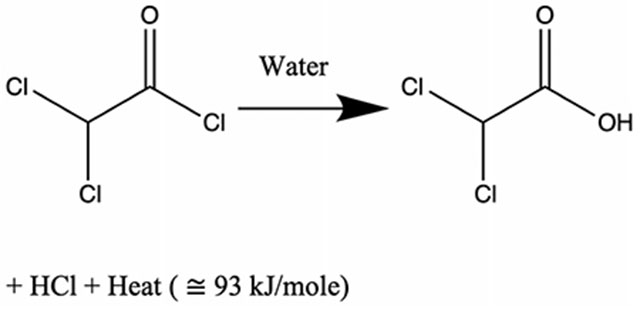


The amount of exothermic energy released, $h_{DCA}\;\left[\frac{kJ}{mole}\right]$, is determined by the chemical species.

Tracking each byproduct individually will be considered in future efforts. Here we assume that the blood provides any buffers needed for reactions as well as acts as a reservoir for byproducts. Neutralization reactions are known to occur on the time scale of 1*e*^−11^ [*s*] and are effectively instantaneous [37]. However, within this approach, DCACl is dissolved in an inert solvent (mineral oil) to delay the hydrolysis by a time constant, $\gamma \;\left[\frac{1}{s}\right]$. Upon its escape, hydrolysis and secondary reactions effectively occur simultaneously and react completely with the latent buffers, and the byproducts return to the blood. Secondary reactions include DCA interactions with the latent tissue buffers, such as bicarbonate or the amide groups within the tissue protein structures. These secondary interactions release energy in the form of heat, $h_{\text{salt}}\;\left[\frac{kJ}{mole}\right]$, and create salt and water byproducts that return to the blood and tissue.


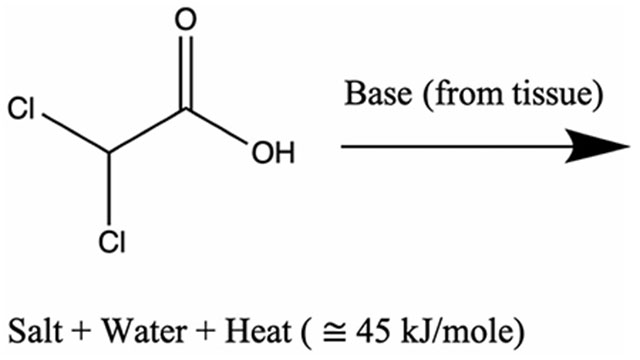


The combined heat released from the initial and secondary reactions, $h\;\left[\frac{kJ}{mole}\right]$, are considered additive, $h=h_{DCA}+h_{\text{salt}}$.

Mass, momentum, and energy transport within this setting are appropriately modeled using a continuum theory of mixtures [25, 26, 28, 38]. Mixture theory allows for each constituent to occupy the same differential volume in space subject to the constraint that the volume fractions, $\varphi_{ı}$, sum to unity:

$$\varphi_{\text{tissue}}+\varphi_{\text{bolus}}+\varphi_{\text{blood}}=1\;\;\forall x\in \Omega$$

Here we will consider the tissue as a fixed porous medium. The porosity, ϕ, represents the ratio of the void volume to the total volume. By convention, within the oil and gas literature, saturation of a given constituent, $s_{ı},ı\in \{blood,bolus\}$, represents the ratio of the void volume filled with the constituent to the total of the void volume in the porous medium. We consider transport of blood, $s_{\text{blood}}$, and the bolus, $s_{\text{bolus}}$, of acid chloride and oily solvent within the pore space:

(1) $$s_{\text{bolus}}+s_{\text{blood}}=1\;\;\forall x\in \text{Ω}$$

Thus, the relationship between volume fractions and porosity is as follows:

$$\varphi_{\text{tissue}}=1-\phi \;\varphi_{\text{bolus}}=\phi s_{\text{bolus}}\;\varphi_{\text{blood}}=\phi s_{\text{blood}}$$

Under these assumptions, our modeling approaches will consider the transport as a two-phase flow within porous media. Pressure, $p_{ı}\in \{blood,bolus\}$, represents an unknown variable for each component. Further, each component is assumed to follow a Darcy flow velocity, $v_{ı}[m/s]$, with permeability $\kappa_{ı}\left[m^{2}\right]$ and viscosity $\mu_{ı}[Pa.s]$. Capillary pressure, $p_{c}$, is empirical and follows a Brooks–Corey model:

(2) $$v_{ı}=-\frac{\kappa_{ı}}{\mu_{ı}}\nabla p_{ı}\;\;p_{c}=p_{\text{bolus}}-p_{\text{blood}}=\frac{p_{d}}{\sqrt{s_{\text{blood}}}}$$

Displacement pressure, $p_{d}$, corresponds to the capillary pressure needed to displace the fluid from the largest pore. Mass balance of the ı-constituent conserves the rate of mass increase within the porous tissue with the convective transport and chemical reactions, $q_{ı}\;\left[\frac{kg}{m^{3}s}\right]$. Each constituent is assumed incompressible with density $\rho_{ı}\left[\frac{kg}{m^{3}}\right]$ :

(3) $$\frac{\partial s_{ı}}{\partial t}+\nabla \cdot \left(s_{ı}v_{ı}\right)=\frac{q_{ı}}{\phi \rho_{ı}}\;\;ı\in \{\text{bolus},\text{blood}\}\;q_{\text{blood}}=-q_{\text{bolus}}=\gamma \rho_{\text{DCACl}}\in \phi s_{\text{bolus}}$$

Chemical reactions provide mass conversion between constituents. Thus, hydrolysis of DCACl is a mass source term for the blood in Equation (3) and corresponds to a mass sink for the bolus. The volume fraction of the bolus occupied by the DCACl is denoted ϵ.

We consider diffusive and convective transport of each constituent’s temperature, $u_{ı}$, resulting from the injected chemical reaction. The temperature rise due to the chemical reaction results from changes in the chemical bond potential energy as it is converted to thermal energy. Temperature changes due to the chemical reactions do not change the total energy of the system [39]:

(4) $$\rho_{ı}c_{ı}\varphi_{ı}\left(\frac{\partial u_{ı}}{\partial t}+v_{ı}\cdot \nabla u_{ı}\right)=\nabla \cdot \left(\varphi_{ı}k_{ı}\nabla u_{ı}\right)+r_{ı}\;ı\in \{\text{tissue},\text{blood},\text{bolus}\}$$

Here, $r_{ı}\;\left[\frac{kJ}{m^{3}\cdot s}\right]$ denotes the energy release with constituent ı by other constituents. The thermal conduction is denoted $k_{ı}$ and the specific heat is denoted $c_{ı}$. Thermal equilibrium is assumed among the tissue, the blood, and the bolus:

$$u_{ı}=u\;\;\forall ı\in \{\text{tissue},\text{blood},\text{bolus}\}$$

The total chemical energy conversion to thermal energy is from the DCACl hydrolysis and secondary reactions:

$$\sum_{ı}r_{ı}=h\gamma M_{\text{DCACl}}(x,t)=h\frac{\gamma \rho_{\text{DCACl}}\epsilon \phi s_{\text{bolus}}}{W_{\text{DCACl}}}$$

$M_{DCACl}\;\left[\frac{mole}{m^{3}\cdot s}\right]$ represents the molarity of the DCACl within the bolus, and the corresponding molecular weight is denoted $W_{\text{DCACl}}\;\left[\frac{kg}{mole}\right]$. Summing the energy balance for the tissue, the blood, and the bolus from (4) yields the governing equation for the temperature change:

(5) $$\rho c\frac{\partial u}{\partial t}+\phi \left(\rho_{\text{blood}}c_{\text{blood}}s_{\text{blood}}v_{\text{blood}}+\rho_{\text{bolus}}c_{\text{bolus}}s_{\text{bolus}}v_{\text{bolus}}\right)\cdot \nabla u=\nabla \cdot (k\nabla u)+h\frac{\gamma \rho_{\text{DCACl}}\epsilon \phi s_{\text{bolus}}}{W_{\text{DCACl}}}$$

For simplicity in notation, k and ρc denote the volume fraction-averaged thermal conduction and thermal inertia, respectively:

$$k\equiv \sum_{ı}\varphi_{ı}k_{ı}\;\;\rho c\equiv \sum_{ı}\varphi_{ı}\rho_{ı}c_{ı}$$

The algebraic relationships for the saturations (1) and pressure (2) are combined with the mass balance (3) and energy balance (5) to yield our governing equations. Here, we choose to describe thermoembolization in terms of temperature, blood pressure, and bolus saturation. Bolus pressure and blood saturation may be recovered from the algebraic relationships in (1) and (2):

(6) Known:ϕ,ϵ,pd,φtissue,γ,h,ρDCACl,WDCACl,κı,μı,ρı,cı,Find:u,pı,sıı∈{blood,bolus}sbolus+sblood=1φtissue+sbolusϕ+sbloodϕ=1λı=sıκıμıvı=−λı∇pıpc=pdsblood=pd1−sbolus=pbolus−pblood−∇⋅((∑ıλı)∇pblood)−∇⋅(λbolus∇pc)=γρDCAClϵϕsbolus(1ρblood−1ρbolus)−ϕ∂sbolus∂t−∇⋅(λblood∇pblood)=γρDCAClϵϕsbolusρbloodρc∂u∂t+ϕ(ρbloodcbloodsbloodvblood+ρboluscbolussbolusvbolus)⋅∇u=∇⋅(k∇u)+hγρDCAClϵϕsbolusWDCACl

The injection pressure, $p_{\text{injection}}$, drives the flow at the tissue-vessel interface, $\partial \Omega_{\text{vessel}}$. The outward normal at the interface is denoted $\vec{n}$. Inflow boundary conditions are assumed at the interface. The bolus enters from the vessels as the only constituent, $s_{0}=1$. The upstream temperature is denoted $u_{0}$ and represents the maximum temperature achieved by the hydrolysis of DCACl:

(7) $$p=p_{\text{injection}}(t),\;\;-\nabla s\cdot \left(\frac{\kappa_{\text{blood}}}{\mu_{\text{blood}}}\nabla p_{\text{blood}}\right)=\left(s_{0}-s\right),\;\;\;\;-\alpha \nabla u\cdot \vec{n}=\left(u_{0}-u\right)\left(\phi \sum_{ı}v_{ı}\right)\cdot \vec{n},\;\;x\in \partial \Omega_{\text{vessel}}$$

Atmospheric pressure, *p*_0_=101.325 kPa = 1 atm, and zero flux are considered on the far boundary, $\partial \Omega_{0}$ :

(8) $$p=p_{0},\;\nabla s_{\text{bolus}}=0,\;\nabla u=0,\;x\in \partial \Omega_{0}$$

Prior to thermoembolization delivery, the experimental setup in this study is in thermal equilibrium with the ambient temperature. The initial temperature is u(x,0)=21°C.

### 2.2. Experimental methods

*Ex vivo* experimental measurements were used to provide an initial validation of our mathematical modeling approach. The experimental methods were described previously [40]. Briefly, experiments were conducted on fresh tissue acquired at necropsy under an Institutional Animal Care and Use Committee-approved protocol. A bolus solution of 2ml DCACl dissolved in mineral oil was delivered into the renal arteries of two kidneys. Specifically, 1ml of 2 mole/L DCACl in oil was sandwiched between 1ml of oil.

MR thermometry was acquired over a 20-min time window to monitor the heat energy release from the exothermic hydrolysis. The time window of the MR thermometry monitoring included both the 25-s thermoembolization injections and subsequent delayed exothermic reactions after the applied pressure had been removed. Thermometry measurements were performed on a 3 T MR scanner (Discovery 750 W, GE Healthcare Technologies, Waukesha, WI) using the body coil for signal excitation and a 32-channel receive-only phased-array cardiac coil. Water proton resonance frequency (PRF) shift measurements were performed *via* a 2D multi-echo fast gradient-recalled echoes (MFGRE) acquisition. Parameters for 2D MFGRE were as follows: time of repetition/first echo time/last echo time = 100/1.50/10 ms; 15 total echoes in 3 ‘shots’ of 5 echoes; echo spacing = 0.604 ms; flip angle = 25°; reconstructed matrix = 256 × 256; field of view = 180 × 180 mm^2^; number of slices = 5; slice spacing and thickness = 5 mm; time per dynamic frame = 22.3 s. The signal-to-noise ratio (SNR) within the corresponding magnitude images was used to estimate the temperature image noise [41]. Signal intensities are sufficient to approximate noise statistics as Gaussian.

### 2.3. Discretization

A finite element method was used to solve the coupled differential Equations (6) for pressure, bolus saturation, and temperature using the *DMPlex* interface of PETSc [42]. The first-order polynomial basis functions were used for the solution field. Backward Euler implicit time discretization was used to propagate the governing equations forward in time. Upwind stabilization [43] was used for numerical stabilization of the convection terms. A Newton line search with cubic backtracking was used to solve the nonlinear system of equations at each time step. A preconditioned generalized minimal residual method (GMRES) iterative solver was used to solve the linear system of equations at each nonlinear iteration. Block–Jacobi preconditioning was used.

The finite-element mesh generation was performed using the imaging data directly. The Amira software [44] was used to manually segment the kidney and large MR-visible vasculature. The image segmentation defines a geometry-conforming surface representation for the boundaries of the solution domain. Facet surfaces representation of the boundary were imported into TetGen [45] for mesh generation. The tetrahedral mesh was adaptively refined near the vessel boundary to both adequately represent the geometry as well as verify convergence of the finite element solution.

A summary of parameters needed to populate our model is provided in Table 1. As thermoembolization is a new technique, literature values of tissue permeability and porosity were imputed as ‘soft rock’ values from the oil and gas literature. The time constant of the hydrolysis, $\gamma \left[\frac{1}{s}\right]$, was estimated directly from a data fit to the MR thermometry data assuming that the temperature, u, reaches a steady state, $u_{ss}$ :

(9) $$u(t)=u_{ss}\left(1-e^{-\gamma t}\right)$$

The upstream temperature, $u_{0}$, was estimated as the maximum temperature achieved in $m_{\text{tissue}}=1\;g$ of tissue subject to the energy release of the exothermic reaction:

$$u_{0}=u(x,0)+\text{Δ}u=u(x,0)+\frac{hn_{\text{DCACl}}}{m_{\text{tissue}}c_{\text{tissue}}}$$

Here, $n_{DCACl}=2e^{-3}$ represents the number of moles of injected DCACl.

## 3. Results

**Figure 2.**
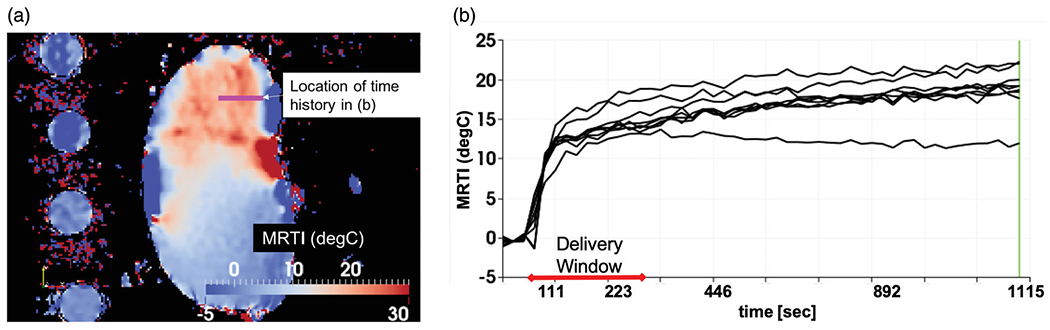
Observed heating. (a) The maximum time point and heating observed in the MR thermometry imaging (MRTI) is shown. Temperature (°C) is shown in relative change from the baseline ambient temperature. (b) The temperature rise is shown as a function of time (seconds) at the spatial positions labeled in (a). For every voxel on the line indicated by the white arrow in (a) there is a time–temperature curve in (b).

For our *ex vivo* model validation, Figure 2 demonstrates the time history of the temperature observed during the thermoembolization procedure. Figure 2(a) shows the maximum time point and heating observed in the MR thermometry. The boundary of the kidney and large vessels are seen in the heating distribution. The slice shown is near the mid-line of the kidney. Temperature is shown in °C relative change from the baseline ambient temperature. Figure 2(b) shows the time temperature history observed at the spatial locations labeled in Figure 2(a). The time window of the delivery is overlaid. The delivery corresponds with the temperature rise. The injection pressure was not applied outside this time window.

**Figure 3.**
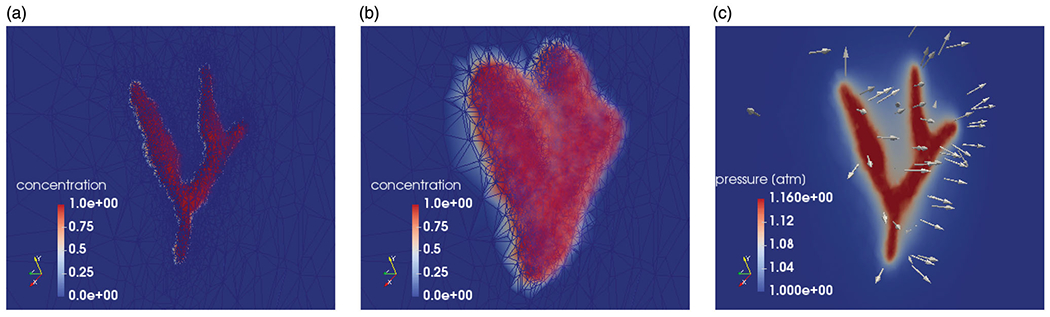
The temporal dynamics of the DCACl solution. Bolus saturation is shown in (a) and (b). The pressure field is shown in (c). The time instance of (a) represents the initial conditions. The final time point of the simulated 20-min experimental time window is shown in (b) and (c). The corresponding predicted temperature field is shown in Figure 4. The color bars in (a) and (b) are provided in color online and represent the volume fraction $s_{\text{bolus}}$. Pressure is shown in atmospheric units, atm.

Results from the image-to-mesh generation pipeline are shown in Figure 3(a). Figure 3(a) illustrates the MR-visible large vessels and the 3D tetrahedralization of this anatomy. The adaptive mesh refinement shown was implemented with the highest resolution near the vessels. The pixel spacing of the image resolution was used as the background mesh to guide the refinement. The final mesh used consisted of 119,068 elements, 82,892 nodes, and 248,676 degrees of freedom for the coupled system. The element size was approximately 1 mm in diameter near the vessel interface.

The simulated solution state variables of bolus saturation, pressure, and temperature are shown in Figure 3. The 3D domain is clipped to view a plane through the renal artery. Both the initial conditions and final time solution of the simulated 20-min experiment are shown. Figure 3(a) and (b) represents the bolus of DCACl and mineral oil flowing from the imaging-visible vessel. The color bars shown represent the volume fraction $s_{\text{bolus}}$. Figure 3(c) represents the pressure field from the corresponding injection pressure. Pressure is shown in atmospheric units, atm. The vector field represents the velocity field from Darcy’s law. The arrows point away from the vessel, indicating the flow direction of the bolus.

**Figure 4.**
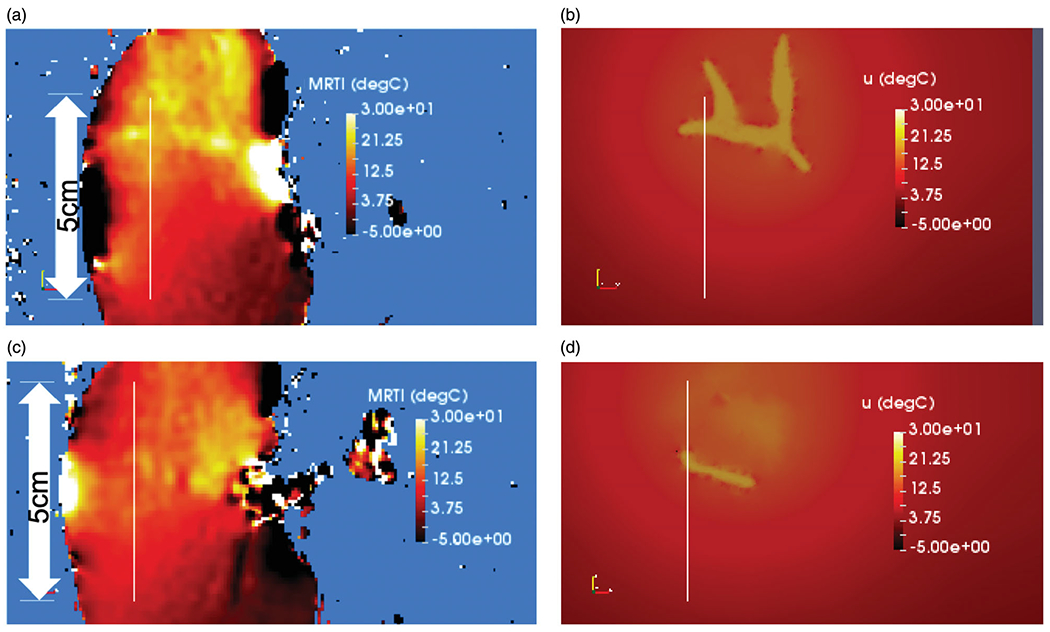
Comparison of simulation to measurement. The final time point of the simulated 20-min experimental time window is shown. (a) Measured and (b) predicted temperatures are shown. The color bars represent relative °C change from the baseline temperature. (a) The measured temperature is shown within a 5 mm imaging plane of the MR thermometry. The maximum temperatures seen occur within the imaging-visible vasculature. Isotherms of the maximum temperatures outline the tissue-vessel boundary, $\partial \Omega_{\text{vessel}}$. (b) The corresponding temperature predictions corresponding to (a) is shown. Convection and diffusion processes at the vessel-tissue interface provide the forcing function for the temperature transport. Similarly, the (c) measured and (d) predicted temperature is shown 1 cm out-of-plane from (a). The length of the line profiles shown in (a)–(d) is 5 mm.

The solution field is interpolated back to the MR thermometry imaging grid in Figure 4 to compare the measured temperature and predicted temperature. The finite element mesh is derived from the surface defined with respect to the imaging grid and is inherently registered to the temperature data. Temperature is shown in °C. The temperature within the vessel is assumed to be the result of hydrolysis. The temperature within the parenchyma represents the convection and diffusion of the heat from the vessel. For this experimental setup, the MR thermometry plane shown in Figure 4(a) is centered on the renal hilum and contains the largest vessels in the kidney. This is the injection and entry point for the acid chloride; the maximum temperature occurs within this imaging plane. Figure 4(c) illustrates the heating 1 cm out-of-plane from Figure 4(a); less heating is seen at this outof-plane location. Figure 4(b,d) is the model predictions corresponding to Figure 4(a,c), respectively.

**Figure 5.**
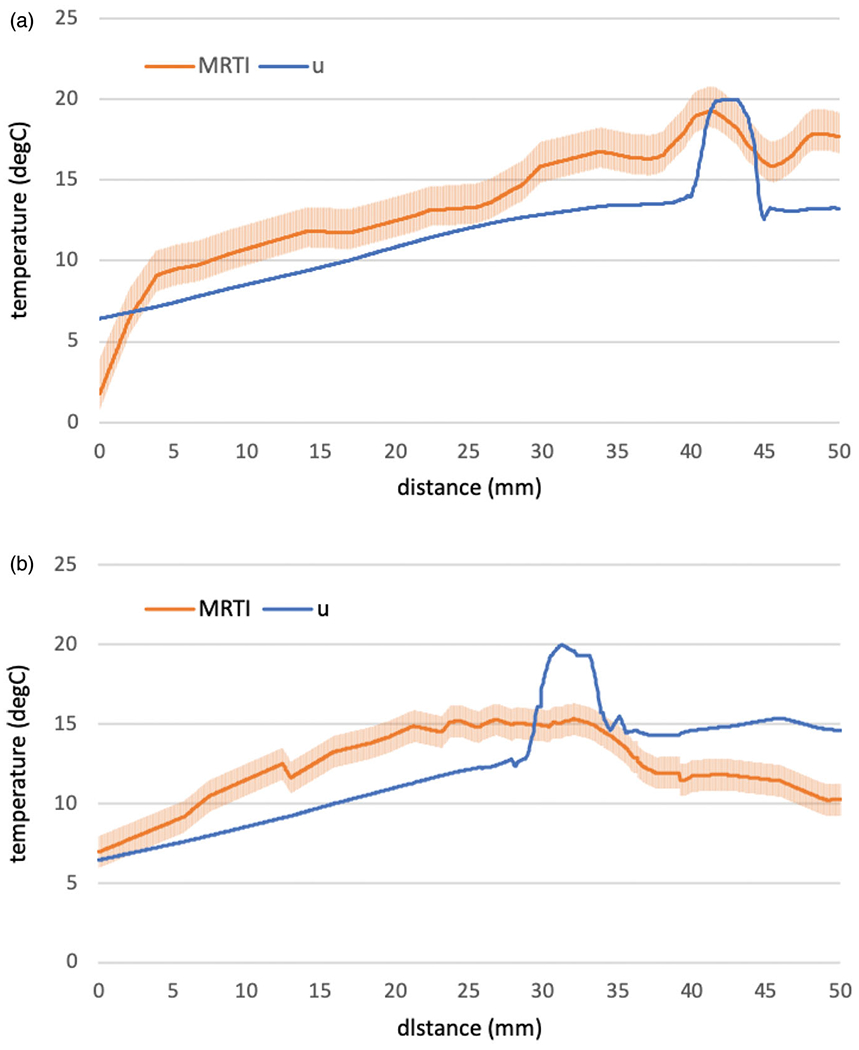
Comparison of simulation to measurement. (a) The predicted and measured temperature is shown as a function of distance [mm] along the 5 cm line profiles illustrated in Figure 4(a) and (b). (b) The predicted and measured temperature is shown as a function of distance [mm] along the 5 cm line profiles illustrated in Figure 4(c) and (d).

The modeled solution field and measured temperature field are compared along a distance versus temperature line profile at the final time of the MR thermometry measurement shown in Figure 5. Measurement error bars are shown in °C. Figure 5(a) corresponds to the line profiles seen in Figure 4(a,b). Figure 5(b) corresponds to the line profiles seen in Figure 4(c,d). Average temperature differences of 2.3 °C (maximum – 4.6 °C) and 2.7 °C (maximum – 4.7 °C) are seen for Figure 5(a,b), respectively. The Pearson correlations between measured and predicted temperatures were calculated as.89 (*p* < .05) for Figure 5(a) and.53 (*p* < .05) for Figure 5(b).

## 4. Discussion

The intent of this work is to demonstrate that the modeling approach described captures the transport phenomena observed during thermoembolization and is likely to be useful in studying the transport mechanisms important for thermoembolization delivery. The heat transfer includes the porous convection of temperature flow from the vessels, diffusive heat flux, as well as a heat source from unreacted DCACl escaping from the mineral oil with respect to the time constant γ. Heat transfer from blood perfusion is not considered in this work but is expected to influence the thermal dose and will be included *in vivo* models of thermoembolization. DCACl concentration is seen to decrease radially from the modeled vessels. This is expected from the inflow boundary conditions, Equation (7), at the tissue-vessel interface. At the vessel interface, the pressure gradient drives the Darcy flow. The inflow conditions maximize the bolus saturation and temperature near the vessels. In particular, inflow boundary conditions for the temperature are reflected in the model predicted temperature rise between 40 and 45 mm of Figure 5(a) and between 30 and 35 mm of Figure 5(b).

The diffusion of the full concentration from the vessel centerline causes heating at the vessel boundary and causes the time delay in heating observed. The notion of ‘transvascular convection’ [53], out-of-plane vessels, and vessels that are not visible on imaging are not directly considered in this approach. Our approach effectively models vessels not visible on imaging as a homogenized transport in the direction normal to the surface of the MR-visible vessels. The approach considers the DCACl reagent in terms of the heat of reaction $h\;\left[\frac{kJ}{mole}\right]$. Additional acidic reactions may be considered by appropriately increasing or decreasing the heat release of the exothermic reaction. Changing the heat of reaction and the injection pressure will allow the study of the competing convective and diffusive properties important to thermoembolization protocol optimization.

Spatial profiles, Figure 5, demonstrated reasonable agreement with the mathematical model and MR thermometry measurements. This initial model development did not achieve pointwise agreement within the measurement error bars. However, results of Pearson correlation analysis indicate that there was a significant positive association between prediction and measurement. The example data shown in Figure 2 provided the intuition for the model development. The observed heating was counterintuitive. The majority of thermal therapy procedures begin to cool immediately upon the end of the delivery window. However, increased heating was observed in the MR thermometry even after the DCACl delivery was complete. The time-temperature history seen in Figure 2(b) indicates that the first-order kinetic modeling, Equation (9), to obtain the hydrolysis time constant γ is appropriate. Further work is needed in cross-validating our approach to evaluate the *a priori* prediction accuracy of our approach. Similar to our previous efforts with other modalities [54], independent calibration and validation datasets are needed to obtain population average model parameters for *a priori* prediction. Additional imaging methods are needed to complement the MR thermometry for validation of the location of the injected bolus of oily solvent and acid chloride.

Importantly, the embolic effects of thermoembolization are not modeled directly in this approach. Within a clinical setting, the artery supplying the tumor will be identified by angiography. The injection velocity of the thermoembolization bolus will be superimposed on the blood flow. Thermal and chemical stress will induce blood coagulation and vessel spasms that embolize the fluid flow and induce ischemic effects. Further model fidelity is needed to incorporate these phenomena. Here, convective transport for our governing Equations (6) stops when the delivery stops and the injection pressure, $p_{\text{injection}}$, is set to zero. The volume of the solution injected directly determines the delivery duration and was chosen based on previous experience of the amount of injected solution before the vessel volume is filled and injection stasis occurs. Conservation of mass requires that the 2 ml injection fills up the vessels and the capillary bed. Only diffusive heating occurs in this model after the delivery window is complete. Convective and diffusive heating only occurs when the injection pressure is non-zero.

Coupling our approach to Navier stokes flow with temperature-dependent viscosity can be considered to model the blood coagulation from the exothermic hydrolysis. Temperature-dependent elasticity properties of the vessels coupled within a fluid structure interface between the blood flow and the vessels may be considered to model the vessel spasms. Coupling our approach with Navier stokes flow and fluid structure interactions would nearly double the computational expense of our approach and are outside the scope of our presented efforts.

Short-term systemic studies document the safety [55] of the thermoembolization approach. In *in vivo* studies, liver enzyme levels did not reveal any acute decompensation, indicating that the procedure is well-tolerated in the animal models. Tissue coagulation achieved by thermoembolization devascularizes the tissue. Recanalization is not expected, and the thermoembolization end products are anticipated to remain at the delivery site. Long-term survival studies are needed to further evaluate safety. Long-term safety studies must incorporate cell damage models to represent the combined cell kill from hyperthermia, chemical denaturation, and ischemia. Traditional Arrhenius and CEM43 thermal damage models [56,57] represent the cell death resulting from the time–temperature history. Novel damage model approaches are needed to account for the coupled chemical and ischemia effects on tissue damage.

In summary, these efforts are a first step toward developing the proposed mathematical approach for guiding parameterization of high-fidelity mathematical models for predicting the thermoembolization acute damage. These predictive models will enable *in silico* experimentation that provides key insight for characterizing fundamental thermal and chemical transport processes needed for control of effective delivery. Virtual experimentation will also guide delivery optimization of convective and diffusive transport processes as this technology progresses toward patient care.

## Figures and Tables

**Table 1.** Parameter summary [46–52].

$k_{\text{blood}}$	$C_{\text{blood}}$	$\rho_{\text{blood}}$	$\mu_{\text{blood}}$	*h*	$\rho_{\text{DCACl}}$	$W_{\text{DCACl}}$	γ	$c_{\text{tissue}}$
$0.53\frac{\text{W}}{m.\text{K}}$	$3600\frac{J}{kg\cdot K}$	$1045\frac{kg}{m^{3}}$	8.90e – 4 Pa s	$138\frac{kJ}{mole}$	$1532\frac{kg}{m^{3}}$	$.147\frac{kg}{mole}$	$2.33\text{e}–3\frac{1}{\text{s}}$	$3600\frac{J}{kg\cdot K}$
$k_{\text{bolus}}$	$c_{\text{bolus}}$	$\rho_{\text{bolus}}$	$\mu_{\text{bolus}}$	ϕ	$p_{\text{injection}}$	$p_{d}$	Permeability	$u_{0}$
$0.15\frac{W}{m\cdot K}$	$1970\frac{J}{kg\cdot K}$	$1280\frac{kg}{m^{3}}$	0.70e – 3 Pa s	.05	1.16atm	0.074atm	$5.\text{e}–12{\text{m}}^{2}$	$98^{{}^{\circ}}C$
